# Continuity of care in UK primary care: a scoping review of measures, challenges, and future interventions

**DOI:** 10.1017/S1463423626101212

**Published:** 2026-05-06

**Authors:** Nikita Kartikapallil, Nicolle Caicedo Arroyave, Jonathan Taylor, Ishveer Sanghera, Mohammad Reduanul Alam, Annabel Lines, Emily Owen-Boukra, Tanya Cohen, Nia Roberts, Victoria Tzortziou Brown, Geoff Wong, Kamal Mahtani, Sophie Park

**Affiliations:** 1 UCL Medical School, University College London, UK; 2 Nuffield Department of Primary Care Health Sciences, University of Oxford, UK; 3 Musgrove Park Hospital, UK; 4 Bodleian Health Care Libraries, University of Oxford, UK; 5 Wolfson Institute of Population Health, Queen Mary University of London, UK

**Keywords:** Continuity, continuity of care, general practice, general practitioner, health policy, healthcare delivery, patient outcomes, primary care

## Abstract

**Background:**

Continuity of care refers to the consistent and coordinated delivery of healthcare services over time. Continuity has been associated with improvements in morbidity and mortality, yet its decline has been identified as a significant concern amid increasing pressures in primary care.

**Aim:**

This review aimed to inform current policy initiatives by synthesizing evidence on how continuity of care is measured, the current challenges faced and proposed future interventions in UK general practice.

**Methods:**

We conducted a literature search for articles published before 15 February 2024, to explore continuity in UK primary care. Screening and data extraction followed PRISMA Scoping Review guidelines, with all studies undergoing double screening to determine eligibility.

**Findings:**

A total of 180 papers were included (95 quantitative, 76 qualitative and 9 mixed-methods). Across the literature, continuity of care was most commonly conceptualized and measured as relational continuity, the Usual Provider of Care (UPC) Index was the most commonly used metric. Informational and managerial continuity were rarely assessed. Certain patient groups, including those with long-term conditions, mental health needs, and multimorbidity, were reported to place greater value on continuity of care. Higher relational continuity was associated with improved patient satisfaction, care coordination and reduced hospital admissions. However, sustaining continuity was frequently challenged by workforce pressures and fragmented information transfer. Although formal and informal interventions to enhance continuity were described, tensions between continuity and access persisted, and continuity was reported to vary across patient groups.

**Conclusion:**

The decline in continuity of care has implications for patient experience and system outcomes. This review highlights the need for system-level approaches and national policy reforms to support continuity, while addressing workforce constraints, access pressures and unequal experiences of care. Further research is needed to evaluate the effectiveness and sustainability of continuity-enhancing interventions and to identify any potential unintended consequences.

## Introduction

Continuity of care (CoC) is a core pillar of primary care, alongside accessibility, comprehensiveness and coordination (Starfield, 1992; Adam and Watson, 2018). More recently, continuity has been defined as ‘*the extent to which a person experiences an ongoing relationship with a clinician and the coordinated clinical care that progresses smoothly as the patient moves between different parts of the health service’* (Couchman *et al.*, 2023). The concept is multifaceted and is interpreted differently by patients, clinicians and policymakers. Haggerty’s widely cited CoC framework delineates three key dimensions: relational, informational, and management continuity (Haggerty *et al.,* 2003; Burch *et al.*, 2024). Relational continuity refers to an ongoing therapeutic relationship between a patient and their healthcare provider, fostering trust and personalized care (Burch *et al.*, 2024). Informational continuity ensures that clinicians and patients have seamless access to medical records, past consultations, and diagnostic results. Managerial continuity, meanwhile, emphasizes coordinated, coherent, and adaptive care over time, particularly for patients with complex or chronic conditions. This framework, while still influential, predates the digital era and does not fully capture the complexities of modern general practice where informational and managerial continuity often blur (Greenhalgh *et al.,* 2022). Emerging perspectives thus propose the concept as being multi-dimensional and more complex.

International evidence suggests that strong continuity is associated with reduced mortality, improved chronic disease management, increased medication adherence, reduced hospital attendance and greater clinician job satisfaction (Guthrie *et al.*, 2008; Gray *et al.,* 2018; Baker *et al.,* 2020). Despite its recognized importance, CoC is under threat. The decline in relational continuity has been identified as one of the most concerning consequences of mounting pressures in general practice (Seddon *et al.*, 2024). The General Practice Patient Survey (GPPS) showed that patients always or almost always/a lot of the time saw their preferred GP 50.2% in 2018, however, this has fallen to 35.4% in 2023 (GP Survey, 2018, 2023). Discontinuity has been linked to poorer treatment adherence, increased hospitalizations, and lower patient satisfaction (Gray *et al.,* 2018). Contributing factors include larger practices, diverse clinical roles and systems focused on rapid access, sometimes at the expense of sustained patient–clinician relationships (Tammes *et al.*, 2017). Alongside these pressures, continuity has been shaped by significant policy and structural changes. Since 2014, initiatives such as the named GP scheme, the introduction of extended access appointments, the development of Primary Care Networks (PCNs), and the more recent shift towards increasing online access and community-based GP-led care have significantly changed how care is organized and delivered (Dineen *et al.*, 2025).

While policy emphasis on CoC is growing, existing reviews are fragmented in scope and outdated in context. One recent scoping review focused on interventions to improve relational continuity, such as personal patient lists or booking systems, based on literature from 2002 onwards. However, it provided limited insight into measurement approaches, outcomes, barriers, or broader care contexts (Fox *et al.*, 2024). Hersch *et al.* outlined the four most common measurement approaches for CoC, however, the discussion was not situated in the UK context and did not address the benefits and challenges associated with each approach (Hersch *et al.,* 2024). A July 2025 systematic review examined the trade-offs between access and continuity, drawing mainly on international studies and without UK-specific focus or attention to wider patient, staff and system outcomes (Goff *et al.,* 2025). Other reviews have examined continuity in the context of care coordination or chronic disease management internationally, but do not capture the full picture of continuity in post-2015 UK primary care, particularly amid NHS reforms and the expansion of PCNs. This period has been characterized by significant structural and policy changes, which have altered how care is organized and delivered. Therefore, to our knowledge, no comprehensive synthesis exists that maps when, and for whom continuity matters, how it is measured, its impacts, the systemic challenges to sustaining it, and which interventions are effective within current UK primary care. This gap highlights the need for a timely, holistic scoping review to inform policy, guide practice, and shape future research.

## Methods

This review aimed to explore key aspects of CoC in primary care, including identifying when it is most critical, the tools and measures used to assess it, its impact on patient care and outcomes, and the major challenges associated with maintaining it. A scoping review design was chosen to provide a rapid summary of research conducted in the last 10 years to inform a more detailed future analysis of continuity outcomes and potential benefits or unintended consequences. The review followed the PRISMA Scoping Review guidelines.

### Identifying the research questions

Our review questions were designed to align with recent and planned NHS policy reforms, including those outlined in the NHS’s Neighbourhood Health Guidelines 2025/26 (NHS England, 2024b). This review aimed to synthesize and critically evaluate the existing literature on continuity of care in UK primary care settings since 2015, addressing the following questions:

1. When does continuity of care matter most in the context of post-2015 UK primary care reforms?
2. What tools and measures are used to assess continuity in primary care?
3. What are the reported improvements to patient care and outcomes?
4. What are the key identified challenges to maintaining continuity of care within post-2015 UK primary care systems?
5. What are the reported interventions aimed at enhancing continuity?

### Identifying relevant studies

We searched Medline (OvidSP), Embase (OvidSP), and CINAHL (EBSCOHost) in 2024, using Medical Subject Headings (MeSH) and free-text terms (see Supplementary Figure 2). Studies were included if published in English between 2015 and 2024. These dates were selected to enable study of the impact of three important changes in the delivery of UK primary care and allow sufficient time for the implementation of these policies and their effects on continuity and related outcomes to be reflected in the published literature. Beginning in 2015, GPs in England have been required to provide every registered patient with a named GP responsible for their care. In 2018, extended access services were introduced to improve patient access to general practice. In 2019, PCNs were introduced as part of the NHS Long-Term Plan. The networks were designed to incentivise general practices to work together in groups covering populations of between 30–50,000 patients (Burch and Whittaker, 2022; Goff *et al.*, 2023; Tammes *et al.*, 2019). Continuity of care has declined over this period and has been the focus of sustained policy and professional concern in UK general practice, further justifying an emphasis on recent structures and incentives (Levene *et al.*, 2024; Murphy and Salisbury, 2020; Pettigrew *et al.,* 2024). The review was restricted to UK-based studies to maintain contextual coherence with the organization of NHS general practice and with UK-specific policy changes. This approach is consistent with other UK-focused syntheses (Fox *et al.*, 2024). Grey literature, including policy reports and bibliographies, were also reviewed (Table 1).

**Table 1.** Inclusion and exclusion criteria

Inclusion criteria	Exclusion criteria
2015 onwards General Practice/Primary Care setting Participants can include any healthcare professional or user within general practice (i.e. not just GPs) UK setting Design/study: qualitative; quantitative; mixed-methods research; and grey literature Includes term ‘Continuity’ (of any type)	Published in or before 2014 Not General Practice/Primary Care setting Not UK setting Main focus of paper is not continuity

### Study selection

Records were de-duplicated and uploaded into the EPPI-reviewer database. Title and abstracts of papers were double screened (NK, RA, IS, NA, AL) against the inclusion criteria. Disagreements between authors were discussed until a consensus was reached through regular data clinics conducted with EO-B and SP.

### Charting the data

A standardized data extraction sheet was developed using Excel and included papers were double-screened at full-text and discrepancies were discussed in data clinics to ensure consistency in collecting key information (see Supplementary Figure 2). This included feedback and adaptations following Patient and Public Involvement (co-applicant TC) to review and refine codes and help develop the study protocol to ensure analysis was relevant to patient and carer perspectives and priorities.

### Collating, summarizing and reporting the results

Emerging findings were reviewed and refined during data clinics involving all members of the research team.

## Results

**Figure 1.**
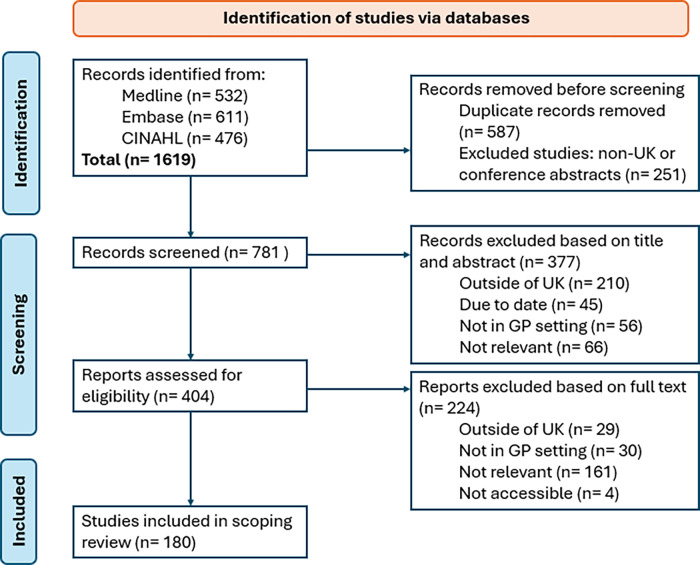
Flowchart of selection of articles for review.

The initial search yielded 1619 papers. 377 studies were excluded based on title and abstract screening and a further 222 were excluded at full-text screening based on our exclusion criteria (Figure 1). 180 papers were included and were fully screened using the data extraction sheet (Supplementary Figure 1). Of these, 95 employed quantitative methodologies, including conceptual, observational, and case studies, as well as reports and opinion pieces. Additionally, 76 studies utilized qualitative approaches, such as systematic reviews, cross-sectional studies, and cohort analyses. 9 studies adopted a mixed-methods design.

The findings presented below apply specifically to UK primary care in the period since 2015, reflecting evidence generated following significant changes in UK health policy. These findings, which are summarized in Table 5, generate new insights regarding the contexts and mechanisms of CoC in primary care.

### Research question 1: When to prioritize continuity of care

Current evidence identifies patient populations who strongly value or prioritize CoC. Patients with long-term conditions (LTCs) that require years of ongoing management such as chronic kidney disease (Brand and Pollock, 2018), and rheumatoid arthritis (RA) (Machin *et al.*, 2017), and those with complex care needs (Rhodes *et al.,* 2016; Greenhalgh *et al.,* 2022; Ladds *et al*., 2023), valued CoC more highly than other patients. Relational continuity was valued in older adults (≥65 years) and patients with multimorbidity (Leniz *et al.*, 2022). Its importance was further amplified for those facing language barriers (Rhodes *et al.*, 2016, Ladds *et al.,* 2023), unstable citizenship status or those who were socioeconomically deprived, with it being more crucial for ethnically diverse groups (Ladds *et al.,* 2023). While the review identified groups that prioritize continuity, none of the included studies provided evidence that clinical benefits were only limited to certain patient groups.

Patients with LTCs wanted their General Practitioner (GP) to be clinically competent, to listen, and take time with them, still demonstrating trust and respect irrespective of whether they have seen them before in situations where continuity was not possible (Murphy and Salisbury, 2020). However, this can contribute to the misconception from practices and patients that ‘any GP will do’, despite evidence that patients who experience CoC usually appreciate its benefits and usually seek it (Gray *et al.,* 2023).

Although several studies identified groups who value continuity, few directly compared preferences across different populations. For example, Machin *et al*., found that poor CoC was perceived as a barrier to discussing mood problems in patients with RA. However, as their study focused exclusively on this group, it remains unclear whether this reliance on continuity is greater than in other conditions. In contrast, Ladds *et al.,* observed that while many patients with long-term or complex conditions prioritize relational continuity with their GP, others, particularly those without complex needs, may be more willing to exchange provider familiarity for timely access or convenience (Greenhalgh *et al.,* 2022; Ladds *et al.,* 2023).

While patient preferences have been shown to vary, studies have also investigated cases in which clinicians have made deliberate efforts to maintain CoC in high-risk cases, such as suspected cancer (Nicholson *et al.,* 2018; Ladds *et al.,* 2023), and vulnerable groups such as those with intellectual disabilities (Thomas *et al.*, 2023). In these cases, GPs took on a heightened role as integrators to try and prevent patients from falling through the gaps in care (Ladds *et al.,* 2023). GPs reinforced this prioritization, emphasizing CoC’s role in managing care transitions (Rhodes *et al.*, 2016) and sensitive consultations involving stigmatized health issues (Rhodes *et al.*, 2016).

### Research question 2: Tools and measures used to assess continuity in primary care

Eleven papers addressed continuity measures (Table 2) that were exclusively concerned with relational continuity and omitted managerial and informational continuity. The field is still evolving, with no consensus on the best measure. Most quantitative studies used whole-population continuity metrics at a practice or patient level, derived from administrative data, rather than measures designed for specific subgroups. The Usual Provider of Care (UPC) Index was most commonly reported, while the GPPS was most used in data analysis.

**Table 2.** Reported measures of continuity of care

*Reported measure of continuity of care*	Description/Formula	Benefits	Challenges	Number of studies that refer to this measure
Usual provider of care (UPC) index	The most commonly reported measure. It measures how often the patient saw the same GP. UPC requires both the number of appointments per patient and a long time frame for accuracy (McDermott *et al.*, 2020) used UPC to investigate ongoing frequent attendance in primary care.	Simple to calculate and widely validated in literature	Unlikely to be used for daily performance management. Minimum number of consultations are required per patient so it is not calculated on all consultations and patients. ‘Does not take into account the total number, frequency or sequence visits’. ‘It does not account for the fact that a patient may have high relational continuity with >1 GP. For example, a patient with five visits to the same GP plus five visits to five different GPs would have the same UPC (0.5) as a patient with 10 visits spread equally across only two GPs.’ (Stafford *et al.*, 2023)	Guthrie *et al.*, 2008; Gray *et al.*, 2018; Ride *et al.*, 2019; Sidaway-Lee *et al.*, 2019; McDermott *et al.*, 2020; Delgado *et al.*, 2022; Gray *et al.*, 2023; Stafford *et al.*, 2023; Jones *et al.*, 2023
Sequential continuity index (SECON)	Measures the proportion of sequential consultations with the same GP.	Verified through peer review	A minimum of two visits is required to calculate	Adam and Watson, 2018; Ride *et al.*, 2019; Delgado *et al.*, 2022
Continuity of care (COC) index, also known as Bice-Boxerman index	This measure captures the different GPs involved in a patient’s care and how many visits occurred to each of them.	Verified through peer review. Often used as a summary statistic.	Did not require patients to be assigned to the same GP. Minimum number of consultations required for a patient to be included (usually 3)	Couchman, 2025; Delgado *et al.*, 2022; Gray *et al.*, 2023; Stafford *et al.*, 2023
St Leonard’s index of continuity of Care (SLICC)	Each month, the number of appointments that patients had with their ‘named’ GP were counted, then divided by the total number of appointments that the same patient has with any GP, expressed as a percentage. Using this index, they identified 57.1% patients had GP appointments with their personal doctor. Over a period of 6 years and an average of 2 appointments a year – 1.5 hrs were spent building a connection with their personal doctor.	Useful to measure CoC regularly, including short term (monthly). Can be quickly interpreted by GPs and staff.	Assumes pre-assigned GPs	Adam and Watson, 2018; Sidaway-Lee *et al.*, 2019; Gray *et al.*, 2023
Own patient ratio (OPR)	Like SLICC, this measure assumes that the patient has a relationship with one particular doctor. However, OPR is calculated by determining how many of the GP’s consultations are with patients who are on their own patient list.	Used in conjunction with SLICC (which included patient perspective), OPR enables GPs to understand how the practice works and allows GPs to make changes so they see their own patients more.	Where patients are allocated a ‘named GP’, this is not an informed choice by the patient (often randomized at the point of registration) and does not include continuity provided by non-clinical staff and therefore ‘may not accurately reflect the patient experience’ ^(23)^	Starfield, 1992; Gray *et al.*, 2023
General practice patient survey (GPPS)	Largest validated survey of patient-reported measures. Annual survey disseminated in January to a selection of patients from every GP practice in England. Information was collated in Burch and Whittaker, 2022, to identify the effectiveness of the national extended access scheme on patient experience and satisfaction. It was concluded that there were no associations. In L’Esperance *et al.*, 2021, they used GPPS to look at the impact of primary care funding on patient satisfaction. In Saunders *et al.*, 2021, they used the GPPS to evaluate that relational continuity between 2011 and 2017 across most socio demographic groups declined.	Captures patient experience and perspectives on a number of aspects of the GP appointment experience	Limited by sample size, heterogeneity and voluntary-response bias. Time consuming and costly method.	Haggerty *et al.*, 2003; L’Esperance *et al.*, 2021; Saunders *et al.*, 2021; Burch and Whittaker, 2022; Gray *et al.*, 2023

### Research question 3: Reported improvements to patient care and outcomes

At a population level, higher CoC has been associated with reduced mortality (Gray *et al.,* 2018; Mahase, 2020), likely due to its role in fostering stronger doctor-patient relationships. Research has also shown that patients exhibit greater trust and comfort when consulting a GP with prior knowledge of their medical history (Turner *et al.,* 2017; Donaghy *et al.*, 2023). It is faster and more efficient to deal with patient problems as successive consultations with the same patients build ‘accumulated knowledge’. Additionally, when GPs have a strong rapport with their patients and clinical responsibilities are clearly defined, practice operations run more smoothly (Gray *et al.,* 2022).

Conversely, patients report reluctance to disclose sensitive information to unfamiliar GPs, often preferring extended wait times to see their preferred clinician (Donaghy *et al.*, 2023). This preference is particularly emphasized when involving high stakes decisions such as DNACPR (Ladds *et al.,* 2023), and mental health concerns (Turner *et al.*, 2017; Donaghy *et al.*, 2023). Patients also seemed to associate longitudinal familiarity with improved diagnostic accuracy, enabling earlier detection of physical and mental health changes (Donaghy *et al.*, 2023), and thereby reducing the perceived risk of misdiagnosis (Rhodes *et al.*, 2016). This relational continuity establishes a therapeutic environment allowing mutual trust, creating psychological safety when sharing sensitive health issues and discussing difficult problems (Nowak *et al.*, 2021).

Patients with multimorbidity found it beneficial to not repeat their complex histories (Engamba *et al.,* 2019), or ‘re-educate and re-inform’ doctors at each appointment (Mason *et al.*, 2016). Doctors identified that continuity facilitated longitudinal assessment of patients’ health status, including monitoring of mobility, cognitive function, speech patterns and overall well-being (McKelvie *et al.*, 2019), thereby supporting decision-making, allowing safer and more efficient practice (Greenhalgh *et al.,* 2022; Ladds *et al.,* 2023). CoC also contributed to enhanced patient perceived validation of health concerns and enhanced shared decision-making with GPs (Nowak *et al.*, 2021).

Strong relational continuity was reported to allow GPs to provide personalized medical recommendations, which may enhance medication adherence and consequently reduce hospitalizations (Barker *et al.*, 2016; Dew and Wilkes, 2018; Nowak *et al.*, 2021; Tammes *et al.*, 2022). Studies demonstrated that patients exhibit greater compliance with treatment regimes and monitoring protocols when relational continuity was present (Tammes *et al.*, 2022), with qualitative research identifying perceived interpersonal accountability as a contributing factor (Turner *et al.*, 2017). Greater CoC was also associated with increased statin prescriptions and improved adherence to statins for secondary prevention in patients aged 30 or older with cardiovascular disease-related conditions (Tammes *et al.*, 2022). Additionally, patients experienced better efficiency of diagnosis and chronic disease management in high CoC scenarios (Nowak *et al.*, 2021).

Patients who were previously frequent attenders had a decreased GP consultation rate after receiving more CoC through a ‘ named GP’ initiative (Barnes *et al.*, 2019), while patients who were unable to see their nominated GP described having to book several appointments for the same problem (Chilton *et al.*, 2021). This fragmented care experience was associated with perceived stigmatization as a ‘nuisance patient’ and subsequent negative psychosocial outcomes, including increased frustration and anxiety (Chilton *et al.*, 2021). Conversely, Goff *et al*. (2024a) found that new working arrangements in Primary Care Networks undermined healthcare professionals’ ability to maintain relational continuity. These changes also caused patients to experience greater inefficiencies when trying to access care.

### Research question 4: Key identified challenges to maintaining continuity of care in modern primary care

Systemic challenges at the primary to secondary care interface, such as delays in information transfer, inadequate referral and discharge communications, inaccessible diagnostic results, fragmented electronic systems, and the unstructured redistribution of tasks between clinicians, represent significant barriers to CoC. These barriers may also arise as consequences of a lack of continuity itself, and a feedback loop is created in which poor continuity contributes to breakdowns in communication and role clarity, which in turn further erode the system’s ability to maintain CoC (Ladds *et al.*, 2023). Maintaining CoC has become increasingly difficult in contemporary health systems (Gray *et al.*, 2023), due to a range of systemic pressures. These include rising workloads, workforce shortages, and growing reliance on locum or part-time staff. Together, these challenges contribute to fragmented care delivery and compromise both patient safety and outcomes. These are significant issues that must be addressed.

The lack of communication between primary and secondary care and other settings of healthcare delivery, has become a major challenge when trying to provide patients with consistent care. Hospital discharge summaries outlining patients’ hospital admissions exhibited suboptimal completion with documentation inconsistencies and significant delays in dissemination (Coyle *et al.*, 2020; Scarfield *et al.,* 2022). This was also highlighted as a challenge in the transition between CAMHS and the adult mental health services where ‘patients fall through the gaps’ (Appleton *et al.,* 2022).

Part‑time working, greater use of allied health professionals, and reliance on locums have all been linked to declining continuity in recent years (McKelvie *et al.*, 2019; Engamba *et al.,* 2019; Khan *et al.,* 2020; Ladds *et al.,* 2023). Locum doctors, often described as ‘professionally isolated’ (Ferguson and Walshe, 2019), have voiced concerns about having access to less information than patients’ regular doctors. McKelvie highlights this issue, stating: ‘*Locum GPs … were very aware of the reduced information that they had in comparison with the patients’ regular doctor.’* (McKelvie *et al.*, 2019: 4). Similarly, newly qualified foundation doctors, who are responsible for producing over 90% of discharge summaries, face challenges in determining what information to include in these letters (McKelvie *et al.*, 2019). Boddy notes: ‘*Over 90% of discharge summaries are authored by newly qualified foundation doctors, who struggle to analyse the information to include and lack insight into the importance of communication for the recipient GP*’ (Boddy *et al.*, 2022: 2). Secondary prevention of Acute Coronary Syndrome is contingent upon strict medication adherence, yet discharge transitions frequently present substantial barriers. These include incomplete or inaccurate medication history, failure to communicate therapeutic modifications, and inadequate information to primary care providers (Goldman and Harte, 2020).

Several systemic challenges to CoC were identified in the literature. Among these were constraints on GP availability (Barnes *et al.*, 2019), with many GPs in portfolio careers working fewer clinical sessions (Saunders *et al.*, 2021). Additionally, maintaining relational continuity was found to impose significant time burdens on GPs, potentially compromising workforce sustainability (Ladds *et al.,* 2023). Further operational barriers included inflexible appointment scheduling systems (Barnes *et al.*, 2019), and inadequate clinical workflow structures (Ladds *et al.,* 2023), both requiring redesign by the practice team to enhance appropriate utilization of electronic health records to facilitate informational continuity (Ladds *et al.,* 2023).

Studies consistently show that some ethnic minority groups experience lower CoC, access and interpersonal care in general practice (Saunders, 2021; Stafford *et al.,* 2023). Indeed, although data from the GPPS show a decline in relational continuity between 2011 and 2017 across most sociodemographic groups, this decline was more pronounced among ethnic minority patients than among those of White ethnicity (Stafford *et al.,* 2023). It has been suggested that these disparities may stem from unequal resource distribution, including underfunded practices and ‘underdoctoring’ in deprived areas where ethnic minorities are overrepresented. (Stafford *et al.,* 2023). The association between ethnicity and continuity, even after controlling for area deprivation, suggests that language barriers and sociocultural norms may create further obstacles and affect whether patients from some ethnic minority groups seek continuity of care (Stafford *et al.,* 2023). Together, these structural and interpersonal factors may undermine CoC and exacerbate health inequalities.

### Research question 5: Reported interventions aimed at enhancing continuity

Tables 3 and 4 summarize the formal and informal interventions used by GP practices to enhance CoC. While formal interventions are embedded in organizational or contractual systems, informal interventions rely on individual or team initiative and are often undocumented. While beneficial these interventions faced implementation challenges and some relied heavily on GP efforts, proving less sustainable. The tension between continuity and accessibility persisted across interventions.

**Table 3.** Formal interventions aimed at enhancing continuity

Strategy/Intervention	Description	Benefits	Challenges	References
Structured admin process & triage	These include systematic monthly reviews by admin staff to identify and notify patients with outstanding abnormal test results through reminder text messages, emails or letters (Nicholson *et al.,* 2018). Admin staff also triage patients ‘case-by-case’ to see whether to offer relational continuity based on information in the medical record or receptionist knowledge of the patient (Ladds *et al.*, 2023), or in some cases the triage is led by GPs, who have stated that familiarity helped the admin work (Ladds *et al.*, 2023).	No information provided.	No information provided.	Nicholson *et al.*, 2018; Ladds *et al.*, 2023
Named GP (NHS contract requirement)	The NHS GMS Contract mandates all GP practices from June 2015 onwards to assign patients a named GP (usually a GP partner) to be responsible for overseeing the care of a patient. Outcome: Lower consultation rate than the control group without an assigned GP and a very small reduction in consultation length (Barnes *et al.*, 2019).	Low-cost, easily-adopted solution that can be easily implemented at the point of registration that can help new patients build rapport and establish continuity with one GP, can help to split a large practice list size between multiple partners, creates accountability for GPs to review hospital correspondence and results from patients on their list	Not integrated into a total-triage approach or appointment booking systems, dependent on GP availability, and patient preferences (Barnes, 2019), does not account for salaried GPs or long-term locum GPs who are also key in CoC can be easily by-passed by adding a read-code to the clinical system, does not address continuity provided by non-clinical staff. Implementation may be an issue ‘Allocating a named GP remains an important first step. The next step is for practices to foster continuity…’ (Gray *et al.*, 2019).	Barnes *et al.*, 2019; Gray *et al.*, 2019; Gray *et al.*, 2023
Personal lists	Single GP accepts long-term responsibility for a list of patients and seeks to empower (inform and encourage self-care) them over time (Gray *et al.*, 2022).	It is quicker and more efficient to respond to patient issues when the GP knows the patient so agreeing management plans is much easier too. Patients do not like having to repeat their story. Personal list systems in general practice improve workload management, reduce stress, and enhance patient care by ensuring continuity with a trusted GP. Switching to personal lists (e.g. 1,400 patients per GP, with ∼100 needing significant care at once) improved workload fairness and doctor morale (Kemple, 2024)	No information provided.	Barnes *et al.*, 2019; Gray *et al.*, 2022; Ladds, 2023; Kemple 2024
Buddy group/micro-teams	Allocating a group of GPs and nurses to a certain patient helped maintain continuity during the shift to telephone consultations.	No information provided.	No information provided.	Engamba *et al.*, 2019; Coombs *et al.*, 2023; Ladds *et al.*, 2023
Collaborative organizational models of general practice	Two models: Close collaboration model (e.g. super-partnerships and multi-site practice organizations): Merge GP contracts and provide joint care services such as managing and identifying illness and referrals to secondary care. Loose collaboration model (e.g. Primary care networks (PCNs) and federations): Retain autonomy but collaborate on out of hours services, diagnostics and other services. Outcomes: Improved MMR (80% to 94%) and flu vaccination rates (+2pp for COPD); care plans for diabetes (10% to 88%) and COPD (53\5 to 87%); pulmonary rehab referrals (45% to 70%).	No information provided.	Slightly lower continuity vs. non-collaborating practices (Forbes *et al.*, 2020)	Forbes *et al.*, 2020; Kovacevic *et al.*, 2023
Shared national electronic records	Key Information Summary (Scotland) shared with out of hours clinicians improved safety, reduced hospital admissions, enhanced clinical empowerment, improved efficiency and communication.	No information provided.	No information provided.	Craig *et al.*, 2015
Shared e-prescribing and records	Continuity-enabled relationships among professionals/patients/carers facilitated medicine access.	No information provided.	No information provided.	Campling *et al.*, 2022
Effective transfer of care management	The effective management of transfer of care was shown to reduce the risk of recurrence and improved patient outcomes.	No information provided.	No information provided.	Goldman *et al.*, 2020
Care coordinators	In the Over 75 Service model, care coordination was managed by practice matrons (senior nurses) rather than GPs, in line with the UK’s goal of delegating such tasks to practice-based staff to free up GP capacity. The matrons ensured managerial continuity by coordinating seamless, consistent care across professionals and relational continuity by serving as the main point of contact for patients, carers, and the care team. This led to reduced service duplication, fewer care gaps, and greater efficiency – supported by the matrons’ dedicated time for the service, a key factor in its success.	No information provided.	No information provided.	Sheaff *et al.*, 2015; MacInnes*et al.*, 2020
Polyclinics	Commissioners aimed at developing a ‘one-stop shop’ offering a comprehensive range of services for the management of long-term conditions and making it easier for patients to obtain a GP appointment.	No information provided.	No information provided.	Sheaff *et al.*, 2015

**Table 4.** Informal interventions aimed at enhancing continuity

Intervention	Description	Benefits	Challenges	References
GP arranged follow up appointments	GP personally arrange follow ups, prioritizing urgent cases and vulnerable patients.	No information provided.	GPs acknowledged that self-booking patients into personal appointment slots was an ‘*effort-intensive preference rather than a workable, system-wide solution’.*	Nicholson *et al.*, 2018; Ladds *et al.*, 2023; Thomas *et al.*, 2023
Patient self booking with GP instruction	Patients are instructed to book follow-ups immediately, either near the follow up date or their discretion.	No information provided.	No information provided.	Nicholson *et al.*, 2018
Ad hoc tracking systems	GPs manually maintain their own personalized lists to track follow-up needs.	No information provided.	No information provided.	Nicholson *et al.*, 2018; Swanepoel, 2020

**Table 5.** Summary of key findings

Research question	Key findings
When continuity matters	Continuity is especially valued by older adults, patients with LTCs, language barriers, or unstable citizenship. Universal approaches remain important because continuity develops cumulatively over time and patient need is dynamic.
Measures of continuity	UPC Index, SECON, Bice-Boxerman (COC), GPPS, SLICC, OPR. Most used: UPC. Each have trade-offs for scalability vs. accuracy.
Impact on outcomes	Improved trust, lower mortality, better medication adherence, reduced hospitalizations, shared decision-making, safer prescribing.
Challenges to continuity	Poor information transfer (especially between primary and secondary care), rising part-time/locum workforce, system-level constraints (e.g. booking systems), digital triage.
Interventions to enhance continuity	Named GP schemes, micro-teams, patient preference policies, workflow redesign, continuity-focused triage.

## Discussion

The evidence base supporting the value of continuity in primary care is burgeoning. However, the concept of continuity is multidimensional, and its meaning varies between patients, clinicians and policymakers (Sheaff *et al.*, 2015). Its implementation must remain adaptable to shifting healthcare needs, patient preferences and systemic constraints to preserve clinical relationships and care quality. Although the evidence supporting CoC is robust, its routine assessment remains insufficiently integrated into clinical practice. The review has focused on literature relating to UK general practice since 2015. This period witnessed significant policy changes in UK primary care. The discussion first considers the impact of these recent policy changes, before reflecting on the five research questions.

Beginning in 2015, UK general practice has undergone three significant national reforms: the named accountable GP scheme (2014/15), universal extended access (2018), and the introduction of PCNs (2019). While a scoping review cannot definitively establish causality, these important shifts have coincided with a continued decline in patient reported CoC, from 50.2% in 2018 to 35.4% in 2023 (GP Survey, 2018, 2023).

Evidence from evaluations of the named accountable GP policy suggests that administratively allocating a GP does not, by itself, produce relational continuity. Tammes *et al*. (2019) found that the scheme was not associated with improvements in either continuity of care or rates of unplanned hospitalization, concluding that more sophisticated interventions are needed beyond mere administrative allocation. This failure has been attributed to the fact that the policy did not guarantee patients would actually see their named clinician at a time of rising GP workloads and part-time working patterns (Couchman *et al.*, 2023).

The introduction of universal extended access was designed to improve appointment availability, particularly for working adults. However, while the scheme improved satisfaction with appointment times for those in full-time employment, it had a negative impact on relational continuity. Mou *et al*. (2025) found that providing these additional access days negatively impacted patients’ experience of continuity of care, though this effect was not linear relative to the number of extra days. This suggests a policy-driven trade-off where prioritizing rapid access frequently occurs at the expense of sustained patient–clinician relationships.

PCNs show mixed effects on continuity. Qualitative evidence indicates that larger-scale care provision can undermine relational continuity by requiring clinicians to work across multiple sites or provide remote consultations for pooled patient lists, leading to ‘fragmented care’ (Goff *et al.*, 2024b). By contrast, recent quantitative data from Odebiyi *et al.* (2025) suggests that larger practice networks and those with pre-existing inter-practice collaborations were actually associated with better continuity. This suggests that while the organizational shift can fragment individual relationships, well-integrated and properly staffed networks may offer a protective structural effect.

In relation to the first research question, the findings indicate that continuity is a priority for vulnerable groups, including patients with LTCs and those facing language barriers or trauma. While the literature identifies certain populations who most value continuity, as noted by Dineen *et al*. (2025), there remains limited empirical evidence demonstrating that these groups derive disproportionately greater clinical benefit. Identification of ‘high-risk’ groups, who are perceived to need continuity most, are sometimes conflated with selective strategies to *only* offer continuity to certain populations. This approach undermines the importance of accumulating continuity over time and the dynamic nature of illness fluctuations and progressions. Indeed, Pahlavanyali *et al*. (2024) note that CoC ‘does not evolve only over certain disease-related consultations and it takes time for doctors to build a relationship with ‘accumulated knowledge’ about their patients’. Similarly, Owen-Boukra *et al.* found that ‘the growth and implementation of cumulative knowledge can flourish through interactions with patients and peers’ (2026). General practice serves universal healthcare needs with longitudinal comprehensive care. Embedding continuity throughout the whole healthcare system is therefore likely to be more successful across time.

The concept of relational injury may be particularly important when considering vulnerable patients. This is the idea that adverse experience or developmental trauma can impair an individual’s ability to engage with healthcare systems. As Burley argues, for those with severe relational injury, CoC is not the context within which care needs to happen, rather it *is* the care that needs to happen (Burley, 2024; Polnay *et al.*, 2023). In this sense, a sustained relationship with a GP could act as a reparative experience, offering the consistency and stability often missing in patients’ lives. This was echoed by a GP involved in the CARE Plus Intervention, who reflected that ‘we are quite often the only person that brings consistency and continuity’ (Mercer *et al.*, 2016). Such perspectives highlight the value of continuity as a form of care that actively addresses psychological and relational needs.

In relation to the second research question, the findings indicate that tools to measure continuity focused on relational continuity, with the UPC most commonly reported and GPPS most frequently used for analysis. While several tools have been developed to measure continuity [Table 2], they often fail to capture qualitative dimensions (e.g. trust, care coordination) or address equity gaps. Certain demographic groups such as ethnic minority groups were found to be notably underrepresented. For example, the 2024 GP survey found that 42% of white patients ‘always/almost always’ accessed their preferred healthcare professional, compared to 34–38% of patients from mixed, Asian, Black, or other ethnicities (NHS England, 2024a). This inequity reflects the Inverse Care Law, first coined by Julian Hart in 1971 who stated ‘The availability of good medical care tends to vary inversely with the need for it in the population served’ (Hart, 1971; 1). As noted in *The Lancet*, positioning this principle at the foreground of policy and healthcare planning could guide efforts to improve health equity and social justice in the coming decades (The Lancet, 2021). Models such as CARE Plus, developed to support patients in areas of high social deprivation, emphasize relational continuity as a core principle (Mercer *et al.*, 2016). Evidence from such interventions demonstrates that continuity is central to delivering high-quality care, particularly for vulnerable groups who are otherwise less likely to experience it.

The third research question concerns CoC and clinical, patient-reported and systems outcomes. CoC has been associated with improved doctor-patient relationships, better medication adherence, lower mortality, reduced hospitalization and the implementation of shared decision-making and personalized care. However, these benefits exist alongside ongoing tensions between sustaining long-term GP-patient relationships and ensuring timely access. While policies designed to expand rapid access, such as the introduction of extended access services, often focus on the ‘supply’ of appointments, GP workforce numbers, and waiting times, they risk unintentionally undermining relational continuity (Fisher *et al.*, 2024). Policy initiatives like Jess’s Rule illustrates the practical consequences of prioritizing rapid access over continuity, highlighting that patients who attend repeatedly with persistent or worsening symptoms benefit when clinicians maintain ongoing relationships and review patterns over time; clinicians are encouraged to ‘reflect, review and rethink’ in these cases (Couchman, 2025). This reinforces the idea that patient demand is not fixed; Owen-Boukra *et al*. (2026) argue that it is dynamic and shaped by the interactions between patients, GPs and practices. When patients feel known, trusted, and ‘looked after’, their sense of uncertainty decreases, and their help-seeking often becomes more measured, reducing the volume and urgency of appointments. The candidacy framework emphasizes how patients recognize their needs, navigate services, and are judged eligible for care (Dixon-Woods *et al.,* 2006). While increasing the permeability of access may facilitate entry into the system, it risks eroding the relational aspects of care that underpin continuity. Striking the right balance remains a central challenge for primary care. The new 10-year plan seeks to address this by implementing neighbourhood health centres. These centres aim to create healthier communities by shifting care from hospitals to local or home settings, focusing on prevention, and leveraging digital solutions to enhance health management, all supporting both accessibility and continuity (NHS England, 2025).

In relation to the fourth research question, the findings highlight the challenges of maintaining CoC, including poor information transfer between primary and secondary care, system-level constraints such as booking systems and digital triage, and the rising use of part-time and locum staff. These barriers may disproportionately affect ethnic minority groups, people living in deprived areas, and those with limited English proficiency or literacy, as longstanding socioeconomic, cultural, language- and disability-related factors, alongside structural and technical limitations, reduce engagement, especially with digital healthcare (Turnbull *et al.*, 2024). This reflects an ‘inverse continuity law’ whereby patients who may have the greatest need are least likely to receive it (Dineen *et al.*, 2025). These practical challenges are further compounded by limitations in how continuity is measured, as the CoC measures identified in this scoping review, and discussed in response to our second research question, focus solely on relational continuity and bi-directional patient-GP interactions, can overlook additional benefits of informational (e.g. review of a discharge letter resulting in a phone call to a patient and subsequent patient advocacy work by the GP) and managerial continuity. Such omissions highlight the importance of broader frameworks, like those proposed by Ladds *et al*. (2023), and the House of Care model (Coulter *et al.,* 2016), which emphasizes teamwork, collaborative and community-focused care to improve outcomes.

While many of the articles identified in this review described part-time working as a significant contributor to the decline in relational continuity, international evidence suggests that clinical hours are not an absolute determinant of a clinician’s ability to provide continuous care. For example, in Norway, where GPs often work clinical sessions only three to four days a week, exceptionally high continuity scores (averaging 85%) are still achieved. This is often facilitated by patients’ willingness to wait for their personal GP for non-urgent matters and the provision of scheduled gaps for non-planned consultations (Sandvik, 2024). Furthermore, a study of Australian GP trainees found that a registrar’s full-time or part-time status had no significant association with the level of continuity they provided to their patients (Pearlman *et al.*, 2016). These findings suggest that with effective practice organization and a patient-centred approach to scheduling, offering high-quality continuity remains feasible within a part-time workforce.

Finally, in relation to the fifth research question, the study identified a range of formal and informal interventions aimed at enhancing continuity. Formal interventions referred to system-wide strategies often embedded in contracts, organizational models, or digital infrastructure. However, informal interventions were characterized as more locally developed, ad hoc practices led by individual GPs or teams which were not formally mandated and often relied on personal initiative. This distinction was also noted in a scoping review which found that, for many GP practices, CoC is delivered as part of routine day-to-day work and often goes undocumented, and is rarely captured in the existing literature (Fox *et al.*, 2024). There seems to be a place for both formal and informal CoC as health systems are complex, and no single approach is likely to be sufficient. The future of continuity interventions may lie in integrating these approaches, ensuring that national frameworks create space and support for local innovations while minimizing reliance on individual effort. Embedding evaluation within such interventions will also be crucial to understand not only their effectiveness, but also their unintended impacts.

### Implications for policy and practice

Recent UK policy reforms have yielded varied results: while the named GP scheme had no measurable impact on continuity, universal extended access has often exacerbated discontinuity, and PCNs have shown mixed effects depending on their internal integration. Strengthening CoC therefore requires system-wide commitment alongside other primary care priorities. Policymakers should align financial incentives to reward care coordination, shifting focus from volume-driven metrics to patient-centred outcomes. Indeed, in February 2024 NHS England announced that the GP Contract Regulations would be amended to explicitly require continuity of care to be considered when deciding how best to respond to patient enquiries (NHS England, 2024c.) Clinically, professional training should emphasize the value of relationships as a core expectation. Researchers must examine digital tools, assessing both their potential to enhance and disrupt CoC (e.g. unintended consequences, such as inequalities in access and experience) (Dineen *et al.*, 2025). Combined, these measures could rebalance primary care toward responsive and proactive, relationship-based care. Within the UK, England has seen the recent introduction of the ‘Ten Year Plan’. This emphasizes the need to shift towards digital, preventive and community-based care (NHS England, 2025). This policy offers a key opportunity in general practice to embed universal approaches to CoC across relational, informational and management systems. Research is crucial to explore the impact these changes have on patients, staff and equitable provision of care.

### Limitations

This review included only English-language studies published after 2015 and focused on the UK, possibly excluding relevant interventions from other healthcare systems. Additionally, the included studies did not allow disaggregation by CoC type (relational, informational, managerial), limiting insights into targeted interventions. This review draws on a wide range of literature that varied in methodological approaches. As studies are not homogenous, our analysis attends to the commonalities and differences across papers rather than seeking to make direct comparison. The diversity of sources was necessary to capture the breadth of current work on CoC and to reflect the complexity of the topic.

### Future research directions

Future studies should measure and understand CoC across all dimensions, including relational, informational, and managerial continuity, as well as qualitative factors such as trust and coordination. Equity-focused research is needed to address disparities among ethnic minority and socially deprived populations, for example exploring the bi-directional work of CoC (e.g. addressing patient needs for advocacy and gate-opening, alongside responding to patient requests and preferences) A realist review framework, for example, can guide exploration of who benefits from relational continuity and under what conditions (Tzortziou Brown *et al.*, 2025).

While this review prioritized recent UK policy impacts, future evidence syntheses should extend the search period prior to 2015 to capture long-term historical trends that predate current regulatory frameworks. Furthermore, researching structurally similar international primary care systems is recommended to help distinguish universal challenges in delivering continuity from those driven specifically by NHS funding and policy levers. Cross-national registry studies and international qualitative syntheses could provide useful methodological templates. Combining population-level administrative data with in-depth qualitative work can both quantify impacts and explain mechanisms. Adopting such mixed, comparative designs would help to identify which continuity-enhancing interventions are transferable across systems and which require local adaptation (Coombs *et al.*, 2023; Nowak *et al.,* 2021; Pahlavanyali *et al.,* 2024).

This review highlights the importance of inclusion, as CoC may not be experienced equally. Current studies, however, provide limited analysis of the underlying mechanisms driving these disparities. Future research should examine how structural, organizational and interpersonal factors contribute to ethnic variation in continuity and should prioritize designs that can detect and explain these inequities.

Further investigation of digital and hybrid care models is warranted, including their impact on continuity and health inequalities. Longitudinal and cross-national studies can clarify sustainability and transferability of interventions. Research should also examine systemic and workforce influences, such as incentives, team models, and working patterns, and the patient perspective, particularly for those with multimorbidity, mental health needs, or relational injury, to strengthen continuity and improve outcomes in primary care.

## Conclusions

Continuity of care remains a cornerstone of effective general practice, but its disruption under ongoing NHS pressures necessitates urgent action. This study highlights reported measures and interventions, particularly integrated solutions that combine workforce redesign, digital integration, and patient-centred metrics, as potential pathways forward. Future research should explore how, why, and under what conditions these interventions may or may not enhance practice. Additionally, thorough evaluation is needed to assess the feasibility and impact of these strategies on continuity, equitable access, workforce sustainability and overall patient health outcomes in real-world settings.

## Supporting information

10.1017/S1463423626101212.sm001 Kartikapallil et al. supplementary material Kartikapallil et al. supplementary material

## Supplementary material

The supplementary material for this article can be found at https://doi.org/10.1017/S1463423626101212.

## References

[ref1] Adam R and Watson E (2018) The role of primary care in supporting patients living with and beyond cancer. Current Opinion in Supportive and Palliative Care 12(3), 261–267. 10.1097/SPC.0000000000000369 30074923

[ref2] Appleton R , Loew J and Mughal F (2022) Young people who have fallen through the mental health transition gap: A qualitative study on primary care support. The British Journal of General Practice 72(719), e413–e420. 10.3399/BJGP.2021.0678 35504728 PMC9090175

[ref3] Baker R , Freeman GK , Haggerty JL , Bankart MJ and Nockels KH (2020) Primary medical care continuity and patient mortality: A systematic review. British Journal of General Practice 70(698), e600–e611. 10.3399/bjgp20X712289 PMC7425204 32784220

[ref4] Barker I , Lloyd T and Steventon A (2016) Effect of a national requirement to introduce named accountable general practitioners for patients aged 75 or older in England: Regression discontinuity analysis of general practice utilisation and continuity of care. BMJ Open 6(9), e011422. 10.1136/bmjopen-2016-011422 PMC5030554 27638492

[ref5] Barnes RK , Cramer H , Thomas C , Sanderson E , Hollinghurst S , Metcalfe C , Jackson S , Record C , Thorley H and Kessler D (2019) A consultation-level intervention to improve care of frequently attending patients: A cluster randomised controlled feasibility trial. BJGP Open 3(1), bjgpopen18X101623. 10.3399/bjgpopen18X101623 PMC6480855 31049406

[ref6] Boddy N , Barclay S , Bashford T and Clarkson PJ (2022) How can communication to GPs at hospital discharge be improved? A systems approach. BJGP Open 6(1), BJGPO.2021.0148. 10.3399/BJGPO.2021.0148 PMC8958742 34620598

[ref7] Brand S and Pollock K (2018) How is continuity of care experienced by people living with chronic kidney disease? Journal of Clinical Nursing 27(1–2), 153–161. 10.1111/jocn.13860 28425171

[ref9] Burch P and Whittaker W (2022) Exploring the impact of the national extended access scheme on patient experience of and satisfaction with general practice: An observational study using the English GP Patient Survey. BJGP Open 6(2), BJGPO.2022.0013. 10.3399/BJGPO.2022.0013 35193885 PMC9447322

[ref8] Burch P , Whittaker W , Bower P and Checkland K (2024) Has the NHS national extended access scheme delivered its policy aims? A case study of two large scale extended access providers. Journal of Health Services Research & Policy 29(3), 191–200. 10.1177/13558196231216657 37978850 PMC11151702

[ref10] Burley A (2024) Relational injury & relational access to healthcare. Enlighten, 28 November. Available at https://www.enlighten.scot/nhs2048/relational-injury-relational-access-to-healthcare-adam-burley/ (accessed 11 March 2026).

[ref11] Campling N , Birtwistle J , Richardson A , Bennett MI , Meads D , Santer M and Latter S (2022) Access to palliative care medicines in the community: An evaluation of practice and costs using case studies of service models in England. International Journal of Nursing Studies 132, 104275. 10.1016/j.ijnurstu.2022.104275 35667146

[ref12] Chilton F , Bradley E and Mitchell T (2021) ‘Lost time’. Patients with early inflammatory/rheumatoid arthritis and their experiences of delays in Primary Care. Musculoskeletal Care 19(4), 495–503. 10.1002/msc.1546 33720502

[ref13] Coombs C , Cohen T , Duddy C , Mahtani KR , Owen E , Roberts N , Saini A , Foster AS and Park S (2023) Primary care micro-teams: An international systematic review of patient and healthcare professional perspectives. British Journal of General Practice 73(734), e651–e658. 10.3399/BJGP.2022.0545 PMC10428005 37549994

[ref15] Couchman E (2025) Jess’s Rule: Advocating for continuity, ownership, and generalism in general practice. British Journal of General Practice 75(760), 500–501. 10.3399/BJGP.2025.0632 PMC12745628 41167968

[ref14] Couchman E , Ejegi-Memeh S , Mitchell S and Gardiner C (2023) Facilitators of and barriers to continuity with GPs in primary palliative cancer care: A mixed-methods systematic review. Progress in Palliative Care 31(1), 18–36. 10.1080/09699260.2022.2074126

[ref16] Coulter A , Kramer G , Warren T and Salisbury C (2016) Building the House of Care for people with long-term conditions: The foundation of the House of Care framework. The British Journal of General Practice 66(645), e288–e290. 10.3399/bjgp16X684745 27033503 PMC4809714

[ref17] Coyle C , Studd L , ElSiofy A and Gelfer Y (2020) Response to: An audit of discharge summaries from secondary to primary care. Irish Journal of Medical Science (1971 -) 189(2), 639–639. 10.1007/s11845-019-02122-4 31673868

[ref21] Dew R and Wilkes S (2018) Attitudes, perceptions, and behaviours associated with hospital admission avoidance: A qualitative study of high-risk patients in primary care. British Journal of General Practice 68(672), e460–e468. 10.3399/bjgp18X697493 PMC6014402 29866709

[ref22] Dineen M , Engamba S , Khan NF , Sidaway-Lee K , Duncan P , Watson J , Evans P and Pereira Gray D (2025) Delivering relational continuity of care in light of recent GP contract changes. British Journal of General Practice 75(757), 342–344. 10.3399/BJGP.2025.0199 PMC12729037 40744755

[ref23] Dixon-Woods M , Cavers D , Agarwal S , Annandale E , Arthur A , Harvey J , Hsu R , Katbamna S , Olsen R , Smith L , Riley R and Sutton AJ (2006) Conducting a critical interpretive synthesis of the literature on access to healthcare by vulnerable groups. BMC Medical Research Methodology, 6(1), 35. 10.1186/1471-2288-6-35 16872487 PMC1559637

[ref24] Donaghy E , Sweeney K , Henderson D , Angus C , Cullen M , Hemphill M , Wang HHX , Guthrie B and Mercer SW (2023) Primary care transformation in Scotland: A qualitative study of GPs’ and multidisciplinary team members’ views. The British Journal of General Practice 74(738), e702–e708. 10.3399/BJGP.2023.0437 PMC10756001 38154939

[ref25] Engamba SA , Steel N , Howe A and Bachman M (2019) Tackling multimorbidity in primary care: Is relational continuity the missing ingredient? The British Journal of General Practice 69(679), 92–93. 10.3399/bjgp19X701201 30705019 PMC6355272

[ref26] Ferguson J and Walshe K (2019) The quality and safety of locum doctors: A narrative review. Journal of the Royal Society of Medicine 112(11), 462–471. 10.1177/0141076819877539 31710823 PMC6851536

[ref27] Fisher R , Beech J , Alderwick H , Price E , Ansari A , Dixon-Woods M and Sinnott C (2024) Rethinking access to general practice: It’s not all about supply. [online] The Health Foundation. Available at https://www.health.org.uk/reports-and-analysis/briefings/rethinking-access-to-general-practice-it-s-not-all-about-supply (accessed 5 September 2025).

[ref28] Forbes LJ , Forbes H , Sutton M , Checkland K and Peckham S (2020) Changes in patient experience associated with growth and collaboration in general practice: Observational study using data from the UK GP Patient Survey. British Journal of General Practice 70(701), e906–e915. 10.3399/bjgp20X713429 PMC7643819 33139333

[ref29] Fox MN , Dickson JM , Burch P , Hind D and Hawksworth O (2024) Delivering relational continuity of care in UK general practice: A scoping review. BJGP Open 8(2), BJGPO.2024.0041. 10.3399/BJGPO.2024.0041 PMC11300996 38438196

[ref30] Goff M , Hindi A , Hammond J and Jacobs S (2025) Access or continuity: A zero sum game? A systematic review of the literature examining the relationship between access and continuity in primary healthcare. BMC Primary Care 26(1), 202. 10.1186/s12875-025-02860-8 40604407 PMC12217832

[ref31] Goff M , Jacobs S , Hammond J , Hindi A and Checkland K (2024a) Access or continuity: A zero sum game? A systematic review of the literature examining the relationship between access and continuity in primary healthcare. BMC Primary Care 26(1), 202. 10.1111/hex.14032 PMC12217832 40604407

[ref89] Goff M , Jacobs S , Hammond J , Hindi A and Checkland K (2024b) Investigating the impact of primary care networks on continuity of care in English general practice: Analysis of interviews with patients and clinicians from a mixed methods study. Health Expectations 27(2), e14032. 10.1111/hex.14032 38556844 PMC10982586

[ref32] Goldman JD and Harte FM (2020) Transition of care to prevent recurrence after acute coronary syndrome: The critical role of the primary care provider and pharmacist. Postgraduate Medicine 132(5), 426–432. 10.1080/00325481.2020.1740512 32207352

[ref36] Gray DJP , Sidaway-Lee K , White E , Thorne A and Evans PH (2018) Continuity of care with doctors – a matter of life and death? A systematic review of continuity of care and mortality. BMJ Open 8(6), e021161. 10.1136/bmjopen-2017-021161 PMC6042583 29959146

[ref34] Gray DP , Sidaway-Lee K and Evans P (2022) Continuity of GP care: Using personal lists in general practice. The British Journal of General Practice 72(718), 208–209. 10.3399/bjgp22X719237 35483941 PMC11189035

[ref33] Gray DP , Sidaway-Lee K , Evans P and Harding A (2019) Having a named doctor in general practice is not enough to improve continuity of care. British Medical Journal 367, l6106. 10.1136/bmj.l6106 31649068

[ref35] Gray DP , Sidaway-Lee K , Johns C , Rickenbach M and Evans PH (2023) Can general practice still provide meaningful continuity of care? British Medical Journal 383, e074584. 10.1136/bmj-2022-074584 37963633

[ref37] Greenhalgh T , Shaw SE , Alvarez Nishio A , Byng R , Clarke A , Dakin F , Faulkner S , Hemmings N , Husain L , Kalin A , Ladds E , Moore L , Rosen R , Rybczynska-Bunt S , Wherton J and Wieringa S (2022) Remote care in UK general practice: Baseline data on 11 case studies. NIHR Open Research 2, 47. 10.3310/nihropenres.13290.2 36814638 PMC7614213

[ref38] Guthrie B , Saultz JW , Freeman GK and Haggerty JL (2008) Continuity of care matters. British Medical Journal 337(aug07 1), a867. 18687724 10.1136/bmj.a867

[ref39] Haggerty JL , Reid RJ , Freeman GK , Starfield BH , Adair CE and McKendry R (2003) Continuity of care: A multidisciplinary review. British Medical Journal 327(7425), 1219–1221. 10.1136/bmj.327.7425.1219 14630762 PMC274066

[ref40] Hart JT (1971) The inverse care law. The Lancet 297(7696), 405–412. 10.1016/S0140-6736(71)92410-X 4100731

[ref41] Hersch D , Klemenhagen K and Adam P (2024) Measuring continuity in primary care: How it is done and why it matters. Family Practice 41(1), 60–64. 10.1093/fampra/cmad122 38160391

[ref42] Jones HE , Anand A , Morrison I , Hurding S , Wild SH , Mercer SW and Shenkin SD (2023) Impact of MidMed, a general practitioner-led modified comprehensive geriatric assessment for patients with frailty. Age & Ageing 52(3), afad006. 10.1093/ageing/afad006 36947740 PMC10032632

[ref43] Kemple TJ (2024) A move to personal lists in general practice will provide continuity of care for patients. British Medical Journal 384, q107. 10.1136/bmj.q107 38228352

[ref44] Khan N , Rudoler D , McDiarmid M and Peckham S (2020) A pay for performance scheme in primary care: Meta-synthesis of qualitative studies on the provider experiences of the quality and outcomes framework in the UK. BMC Family Practice 21(1), 142. 10.1186/s12875-020-01208-8 32660427 PMC7359468

[ref45] Kovacevic L , Naik R , Lugo-Palacios DG , Ashrafian H , Mossialos E and Darzi A (2023) The impact of collaborative organisational models and general practice size on patient safety and quality of care in the English National Health Service: A systematic review. Health Policy 138, 104940. 10.1016/j.healthpol.2023.104940 37976620

[ref46] L’Esperance V , Schofield P and Ashworth M (2021) The provision of additional services in primary care: A cross-sectional study of incentivised additional services, social deprivation, and ethnic group. BJGP Open 5(1), bjgpopen20X101141. 10.3399/bjgpopen20X101141 PMC7960517 33199308

[ref47] Ladds E , Greenhalgh T , Byng R , Rybczynska-Bunt S , Kalin A and Shaw S (2023) A contemporary ontology of continuity in general practice: Capturing its multiple essences in a digital age. Social Science & Medicine 332, 116112. 10.1016/j.socscimed.2023.116112 37535988

[ref49] Leniz J , Gulliford M , Higginson IJ , Bajwah S , Yi D , Gao W and Sleeman KE (2022) Primary care contacts, continuity, identification of palliative care needs, and hospital use: A population-based cohort study in people dying with dementia. The British Journal of General Practice, 72(722), e684–e692. 10.3399/BJGP.2021.0715 35817583 PMC9282808

[ref50] Levene LS , Baker RH , Newby C , Couchman EM and Freeman GK (2024) Ongoing decline in continuity with GPs in English General Practices: A longitudinal study across the COVID-19 pandemic. Annals of Family Medicine 22(4), 301–308. 10.1370/afm.3128 38914438 PMC11268676

[ref51] Machin A , Hider S , Dale N and Chew-Graham C (2017) Improving recognition of anxiety and depression in rheumatoid arthritis: A qualitative study in a community clinic. British Journal of General Practice 67(661), e531–e537. 10.3399/bjgp17X691877 PMC5519124 28716999

[ref52] MacInnes J , Baldwin J and Billings J (2020) The over 75 service: Continuity of integrated care for older people in a United Kingdom primary care setting. International Journal of Integrated Care 20(3), 2. 10.5334/ijic.5457 PMC7366863 32742248

[ref53] Mahase E (2020) Mortality rates are lower with higher continuity of care, review finds. British Medical Journal 370, m3184. 10.1136/bmj.m3184 32784197

[ref54] Mason B , Nanton V , Epiphaniou E , Murray S A , Donaldson A , Shipman C , Daveson B A , Harding R , Higginson I J , Munday D , Barclay S , Dale J , Kendall M , Worth A and Boyd K (2016) ‘My body‘s falling apart.’ Understanding the experiences of patients with advanced multimorbidity to improve care: Serial interviews with patients and carers. BMJ Supportive & Palliative Care 6(1), 60–65. 10.1136/bmjspcare-2013-000639 25023218

[ref55] McDermott A , Sanderson E , Metcalfe C , Barnes R , Thomas C , Cramer H and Kessler D (2020) Continuity of care as a predictor of ongoing frequent attendance in primary care: A retrospective cohort study. BJGP Open 4(5), bjgpopen20X101083. 10.3399/bjgpopen20X101083 PMC7880190 33051221

[ref56] McKelvie S , Moore A , Croxson C , Lasserson DS and Hayward GN (2019) Challenges and strategies for general practitioners diagnosing serious infections in older adults: A UK qualitative interview study. BMC Family Practice 20(1), 56. 10.1186/s12875-019-0941-8 31027482 PMC6486693

[ref57] Mercer SW , O’Brien R , Fitzpatrick B , Higgins M , Guthrie B , Watt G and Wyke S (2016) The development and optimisation of a primary care-based whole system complex intervention (CARE Plus) for patients with multimorbidity living in areas of high socioeconomic deprivation. Chronic Illness 12(3), 165–181. 10.1177/1742395316644304 27068113 PMC4995497

[ref58] Mou L , Lau Y-S , Burch P and Whittaker W (2025) Did a national extended access scheme translate to improvements in patient experience to GP services in England? A retrospective observational study using patient-level data from the English GP patient survey. BMC Health Services Research 25, 355. 10.1186/s12913-025-12447-9 40055699 PMC11889905

[ref59] Murphy M and Salisbury C (2020) Relational continuity and patients’ perception of GP trust and respect: A qualitative study. British Journal of General Practice 70(698), e676–e683. 10.3399/bjgp20X712349 PMC7425201 32784221

[ref60] NHS England (2024a) GP patient survey results 2024. [online] NHS England. Available at https://www.gov.uk/government/statistics/gp-patient-survey-results-2024 (accessed 8 September 2025).

[ref61] NHS England (2024b) Neighbourhood health guidelines [online] NHS England. Available at https://www.england.nhs.uk/long-read/neighbourhood-health-guidelines-2025-26/ (accessed 8 September 2025).

[ref62] NHS England (2024c) Arrangements for the GP contract in 2024/25. [online] NHS England. Available at https://www.england.nhs.uk/publication/arrangements-for-the-gp-contract-in-2024-25/ (accessed 11 March 2026).

[ref63] Nicholson BD , Goyder CR , Bankhead CR , Toftegaard BS , Rose PW , Thulesius H , Vedsted P and Perera R (2018) Responsibility for follow-up during the diagnostic process in primary care: A secondary analysis of International Cancer Benchmarking Partnership data. British Journal of General Practice 68(670), e323–e332. 10.3399/bjgp18X695813 PMC5916079 29686134

[ref64] Nowak DA , Sheikhan NY , Naidu SC , Kuluski K and Upshur RE (2021) Why does continuity of care with family doctors matter?: Review and qualitative synthesis of patient and physician perspectives. Canadian Family Physician 67(9), 679–688. 10.46747/cfp.6709679 34521712 PMC9683367

[ref90] Odebiyi B , Gibson J , Goff M , Hindi AM , Hammond J , Checkland K , Sutton M and Jacobs S (2025) Patient perceptions of relational continuity in England: Insights from two cross-sectional surveys. BJGP Open 9(4), BJGPO.2024.0267. 10.3399/BJGPO.2024.0267 40360191 PMC12820509

[ref65] Owen-Boukra E , Burford B , Cohen T , Duddy C , Dunn H , Fadia V , Goodman C , Henry C , Lamb EI , Ogden M , Rapley T , Rees EL , Roberts NW , Royer-Gray E , Vance G , Wong G and Park S (2026) GP workforce sustainability to maximise effective and equitable patient care: A realist review. British Journal of General Practice 76(764), e192–e203. 10.3399/BJGP.2025.0061 PMC13060717 41022521

[ref66] Pahlavanyali S , Hetlevik Ø. , Baste V , Blinkenberg J and Hunskaar S (2024) Continuity and breaches in GP care and their associations with mortality for patients with chronic disease: An observational study using Norwegian registry data. British Journal of General Practice, 74(742), e347–e354. 10.3399/BJGP.2023.021 PMC11044022 38621803

[ref67] Pearlman J , Morgan S , van Driel M , Henderson K , Tapley A , McElduff P , Scott J , Spike N , Thomson A and Magin P (2016) Continuity of care in general practice vocational training: Prevalence, associations and implications for training. Education for Primary Care 27(1), 27–36. 10.1080/14739879.2015.1101871 26862796

[ref68] Pettigrew LM , Petersen I , Mays N and Cromwell D (2024) The changing shape of English general practice: A retrospective longitudinal study using national datasets describing trends in organisational structure, workforce and recorded appointments. BMJ Open 14(8), e081535. 10.1136/bmjopen-2023-081535 PMC11404227 39227175

[ref69] Polnay A , Pugh R , Barker V , Bell D , Beveridge A , Burley A , Lumsden A , Mizen CS and Wilson L (2023) Chapter 15 – Applications of psychodynamic theory and principles outside of specialist psychotherapy settings. In Polnay A , Pugh R , Barker V , Bell D , Beveridge A , Burley A , Lumsden A , Mizen CS and Wilson L (eds), Cambridge Guide to Psychodynamic Psychotherapy. Cambridge University Press, pp. 231–243. 10.1017/9781009104425.017

[ref70] Rhodes P , Campbell S and Sanders C (2016) Trust, temporality and systems: How do patients understand patient safety in primary care? A qualitative study. Health Expectations 19(2), 253–263. 10.1111/hex.12342 25644998 PMC5024004

[ref71] Ride J , Kasteridis P , Gutacker N , Doran T , Rice N , Gravelle H , Kendrick T , Mason A , Goddard M , Siddiqi N , Gilbody S , Williams R , Aylott L , Dare C and Jacobs R (2019) Impact of family practice continuity of care on unplanned hospital use for people with serious mental illness. Health Services Research 54(6), 1316–1325. 10.1111/1475-6773.13211 31598965 PMC6863233

[ref72] Sandvik H (2024) Continuity of care in general practice: The Norwegian experience. British Medical Journal 384, q109. 10.1136/bmj.q109 38228353

[ref73] Saunders CL , Flynn S , Massou E , Lyratzopoulos G , Abel G and Burt J (2021) Sociodemographic inequalities in patients’ experiences of primary care: An analysis of the General Practice Patient Survey in England between 2011 and 2017. Journal of Health Services Research & Policy 26(3), 198–207. 10.1177/1355819620986814 33517786 PMC8182330

[ref74] Scarfield P , Shepherd T D , Stapleton C , Starks A , Benn E , Khalid S , Dayment B , Moate A , Mohamed S and Lee J (2022) Improving the quality and content of discharge summaries on acute medicine wards: A quality improvement project. BMJ Open Quality 11(2), e001780. 10.1136/bmjoq-2021-001780 PMC8991046 35393294

[ref75] Seddon J , Friedrich C , Wadd S , Dicks D , Scott S , Robinson A and Walker C (2024) Improving patient experience for people prescribed medicines with a risk of dependence or withdrawal: Co-designed solutions using experience based co-design. BMC Primary Care 25(1), 17. 10.1186/s12875-023-02253-9 38184517 PMC10770999

[ref76] Sheaff R , Halliday J , Ovretveit J , Byng R , Exworthy M , Peckham S and Asthana S (2015) Integration and continuity of primary care: Polyclinics and alternatives – A patient-centred analysis of how organisation constrains care co-ordination. Health Services and Delivery Research Volume 3(35), 1–148. Chapter 11, Conclusions. Available at https://www.ncbi.nlm.nih.gov/books/NBK311217/26312365

[ref77] Sidaway-Lee K , Gray DP and Evans P (2019) A method for measuring continuity of care in day-to-day general practice: A quantitative analysis of appointment data. British Journal of General Practice 69(682), e356–e362. 10.3399/bjgp19X701813 PMC6478463 30803982

[ref78] Stafford M , Bécares L , Hayanga B , Ashworth M and Fisher R (2023) Continuity of care in diverse ethnic groups: A general practice record study in England. British Journal of General Practice 73(729), e257–e266. 10.3399/BJGP.2022.0271 PMC9639601 36316161

[ref79] Starfield B (1992) Primary Care: Concept, Evaluation, and Policy. Oxford University Press.

[ref80] Survey GP (2018/2023) GP Patient Survey. See how your GP practice is doing or compare practices. [online]. Available at https://www.gp-patient.co.uk/ (accessed 31 August 2025).

[ref81] Swanepoel R (2020) Evaluating the relational continuity of care of four GP practices, one of which uses personalised lists. British Journal of General Practice 70(suppl 1), bjgp20X711713. 10.3399/bjgp20X711713 32554691

[ref83] Tammes P , Payne RA and Salisbury C (2022) Association between continuity of primary care and both prescribing and adherence of common cardiovascular medications: A cohort study among patients in England. BMJ Open 12(9), e063282. 10.1136/bmjopen-2022-063282 PMC9472141 36100300

[ref82] Tammes P , Payne RA , Salisbury C , Chalder M , Purdy S and Morris RW (2019) The impact of a named GP scheme on continuity of care and emergency hospital admission: A cohort study among older patients in England, 2012–2016. BMJ Open 9(9), e029103. 10.1136/bmjopen-2019-029103 PMC6773345 31548353

[ref84] Tammes P , Purdy S , Salisbury C , MacKichan F , Lasserson D and Morris RW (2017) Continuity of primary care and emergency hospital admissions among older patients in England. The Annals of Family Medicine 15(6), 515–522. 10.1370/afm.2136 29133489 PMC5683862

[ref48] The Lancet (2021) 50 years of the inverse care law. Lancet (London, England) 397(10276), 767. 10.1016/S0140-6736(21)00505-5 33640043

[ref85] Thomas N , Atherton H , Dale J , Smith K and Crawford H (2023) General practice experiences for parents of children with intellectual disability: A systematic review. BJGP Open 7(3), BJGPO.2023.0010. 10.3399/BJGPO.2023.0010 37185167 PMC10646198

[ref86] Turnbull J , Prichard J , MacLellan J and Pope C (2024) eHealth literacy and the use of NHS 111 online Urgent Care Service in England: Cross-sectional survey. Journal of Medical Internet Research 26, e50376. 10.2196/50376 38833297 PMC11185907

[ref87] Turner KM , Percival J , Kessler D and Donovan J (2017) Exploring patients’ treatment journeys following randomisation in mental health trials to improve future trial conduct: A synthesis of multiple qualitative data sets. Trials 18(1), 279. 10.1186/s13063-017-2030-4 28619121 PMC5472926

[ref88] Tzortziou Brown V , Park S , Mahtani K , Taylor S , Owen-Boukra E , Taylor J , Richards O , Begum S and Wong G (2025) Implementing relational continuity in general practice – Understanding who needs it, when, to what extent, how and why: A realist review protocol. BMJ Open 15, e104081. 10.1136/bmjopen-2025-104081 PMC12421597 40930545

